# Imaging the Cell Morphological Response to 3D Topography and Curvature in Engineered Intestinal Tissues

**DOI:** 10.3389/fbioe.2020.00294

**Published:** 2020-04-07

**Authors:** Gizem Altay, Sébastien Tosi, María García-Díaz, Elena Martínez

**Affiliations:** ^1^Biomimetic Systems for Cell Engineering, Institute for Bioengineering of Catalonia, The Barcelona Institute of Science and Technology, Barcelona, Spain; ^2^Advanced Digital Microscopy Core Facility, Institute for Research in Biomedicine, The Barcelona Institute of Science and Technology, Barcelona, Spain; ^3^Centro de Investigación Biomédica en Red en Bioingeniería, Biomateriales y Nanomedicina, Madrid, Spain; ^4^Department of Electronics and Biomedical Engineering, University of Barcelona, Barcelona, Spain

**Keywords:** hydrogel scaffold, confocal microscopy, substrate curvature, cell morphology, cell orientation, histological section, small intestine, villus

## Abstract

While conventional cell culture methodologies have relied on flat, two-dimensional cell monolayers, three-dimensional engineered tissues are becoming increasingly popular. Often, engineered tissues can mimic the complex architecture of native tissues, leading to advancements in reproducing physiological functional properties. In particular, engineered intestinal tissues often use hydrogels to mimic villi structures. These finger-like protrusions of a few hundred microns in height have a well-defined topography and curvature. Here, we examined the cell morphological response to these villus-like microstructures at single-cell resolution using a novel embedding method that allows for the histological processing of these delicate hydrogel structures. We demonstrated that by using photopolymerisable poly(ethylene) glycol as an embedding medium, the villus-like microstructures were successfully preserved after sectioning with vibratome or cryotome. Moreover, high-resolution imaging of these sections revealed that cell morphology, nuclei orientation, and the expression of epithelial polarization markers were spatially encoded along the vertical axis of the villus-like microstructures and that this cell morphological response was dramatically affected by the substrate curvature. These findings, which are in good agreement with the data reported for *in vivo* experiments on the native tissue, are likely to be the origin of more physiologically relevant barrier properties of engineered intestinal tissues when compared with standard monolayer cultures. By showcasing this example, we anticipate that the novel histological embedding procedure will have a positive impact on the study of epithelial cell behavior on three-dimensional substrates in both physiological and pathological situations.

## Introduction

Many epithelial tissues exhibit complex morphologies that are mostly dominated by curved surfaces such as those found in lung alveoli and intestinal villi (Torras et al., 2018; Baptista et al., 2019). These three-dimensional (3D) topographies generate gradients in biochemical signals and mechanical tension that play a key role in cell polarization, morphology, and function (Farin et al., 2016; Krndija et al., 2019). While traditionally ignored in flat *in vitro* models, 3D topographies are distinctive elements in the emergence and further development of a new generation of tissue-engineered cell culture substrates (Griffith and Swartz, 2006). These 3D models are capable of capturing the complex physiological responses of tissue *in vitro* and have been employed to systematically study the effects of *in vivo*-like tissue geometry and curvature on cell fate (Griffith and Swartz, 2006; Baptista et al., 2019).

Hydrogels are widely used as scaffold materials for tissue engineering and 3D cell culture applications (Slaughter et al., 2009; Caliari and Burdick, 2016). Due to their porous network structure and soft mechanical properties, hydrogels mimic important elements of the native extracellular matrix. Also, their rich water content facilitates the diffusion of biochemical factors and oxygen and also enables long-term maintenance of mammalian cell cultures (Peppas et al., 2006; DeForest and Anseth, 2012; Caliari and Burdick, 2016). Hydrogels can be engineered with different topographies and can thus be used to reproduce the 3D architecture of native tissues (Khademhosseini and Langer, 2007; Torras et al., 2018). In the case of the small intestine, several strategies have been proposed to fabricate villus-like hydrogels such as replica molding or stereolithography-based 3D printing (Wang et al., 2017; Creff et al., 2019). Using a different approach, we have recently developed a method to fabricate microstructured hydrogel scaffolds mimicking the small intestinal villi by using a photolithography-based microfabrication technique (Castaño et al., 2019). This moldless approach yielded high aspect ratio, finger-like hydrogel microstructures with the physiological shape and dimensions of the native villi. Moreover, not only did these scaffolds support the growth and differentiation of intestinal epithelial Caco-2 cells along the villi but they also induced improved cell polarization and tissue barrier properties compared to the standard monolayer cell cultures. These benefits were attributed to the more physiologically realistic environment provided by the 3D model.

Recently, it has been reported that, *in vivo*, parameters such as cell density and cell anisotropy differ significantly along the crypt-villus axis of the intestinal epithelium (Krndija et al., 2019). This raises the question if analogous effects can also be observed *in vitro* on engineered intestinal tissue models. For that purpose, the optical visualization of individually resolved cells along the vertical axis of 3D villus-like microstructures is required. Confocal fluorescence microscopy is the most widespread imaging technique used in the field of life sciences and enables optical sectioning for *in situ* cell imaging (Graf and Boppart, 2010; Leferink et al., 2016). However, imaging large tissue-engineered constructs with high resolution is technically challenging due to sample thickness and high scattering (Pampaloni et al., 2007). In particular, engineered intestinal tissues contain relatively large finger-like microstructures with a high aspect ratio (∼500 μm in height, ∼100 μm in diameter). As such, due to the working distance restrictions of high magnification objective lenses, the total thickness of the scaffolds (often ∼1000 μm) precludes high-resolution imaging along the whole structure (Smith et al., 2010; Short et al., 2017). To overcome this problem, advanced techniques such as multiphoton microscopy, light sheet microscopy, or optical coherence tomography can be used. However, the sophisticated equipment needed is often not widely available in standard tissue engineering laboratories (Graf and Boppart, 2010; Leferink et al., 2016).

Histological processing is the gold standard for tissue characterization in medical practice and is often available as a support facility at most research centers. When applied to cells grown on 3D tissue-engineered constructs, it can provide crucial information on parameters such as their viability, proliferation, and differentiation. However, due to their high water content, hydrogels are very sensitive to routine histology procedures (James et al., 2004; Yang et al., 2007). In standard paraffin processing, the exchange of tissue water by ethanol, followed by a hydrophobic solvent (e.g., xylene), results in the collapse of the hydrogel scaffolds (Loebsack et al., 1999). Cryosectioning is another standard technique for tissue processing. It is performed at very low temperatures (about −20 to −30°C) after embedding the tissue in a gel-like medium called optimum cutting temperature (OCT). Then again, the water-rich hydrogel samples show the formation of ice crystals within the hydrogels themselves, resulting in sample brittleness and poor cryosection quality, even after infiltration with a 30% sucrose solution for cryoprotection (Ruan et al., 2013). Different strategies have been pursued to improve the sectioning of hydrogel scaffolds. Among them, improved cryosections were obtained by including, as an additional step in the protocol, an overnight incubation with non-protein-based solutions such as polyvinyl alcohol or the same OCT medium that is later used for embedding (Ruan et al., 2013). In another approach, it was recently reported that the integrity of the hydrogels during sectioning was preserved by employing vibrating microtomy, which is commonly used in sectioning soft tissues such as the brain (Short et al., 2017). Nevertheless, despite their potential, these techniques have rarely been applied to the characterization of microstructured hydrogel scaffolds (Sung et al., 2011; Costello et al., 2014b). In fact, our previous attempts to produce histological sections of engineered intestinal tissues by using either cryosectioning or vibrating microtomy resulted in the collapse of the delicate villus-like microstructures.

In this study, we present a new method to preserve complex hydrogel microstructures in histological sections by employing a photopolymerisable polyethylene glycol diacrylate (PEGDA) polymer as the embedding medium. This resulted in hydrogel blocks that were suitable for processing by either cryosectioning or vibrating microtomy and that yielded undistorted sections of the engineered 3D hydrogels. By using this method, the cell morphological response of intestinal epithelial Caco-2 cells to villi-like hydrogel microstructures was successfully evaluated. High-resolution imaging was performed and revealed that cell morphology and nuclear orientation changes significantly along the vertical axis of the villus-like microstructures while also being dramatically impacted by the curvature of the structures. Also, the expression of epithelial polarization markers, such ZO-1, were remarkably higher toward the tips of the villi than at their bases. Altogether, these findings demonstrate that, similar to what has been reported *in vivo*, the complex topographies and curvatures recreated by the engineered *in vitro* models significantly affect cell shape and polarization. As such, this methodology paves the way to gain better insights about the growth of epithelial cells on 3D artificial structures and to study the effects of tissue curvature in physiological events, such as morphogenesis or homeostasis, and pathological situations such as wound healing processes.

## Materials and Methods

### Fabrication of Villus-Like Microstructured Hydrogels and Cell Culture

**FIGURE 1:**
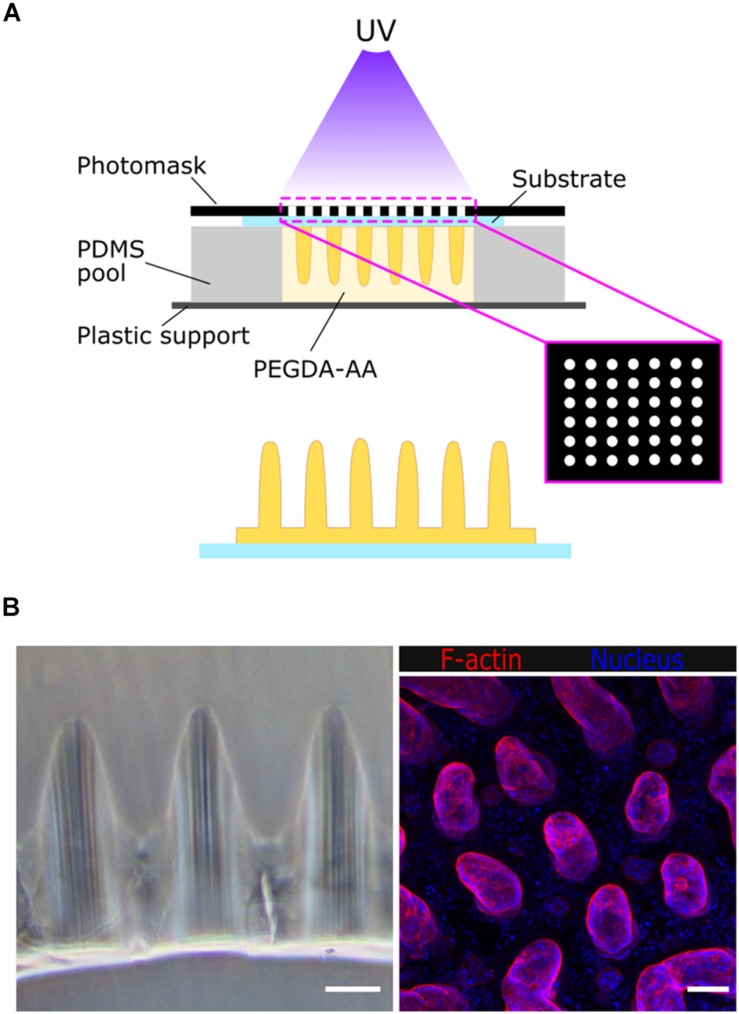
**(A)** Schematic drawing of the fabrication of villus-like microstructured PEGDA-AA hydrogel scaffolds. **(B)** Left: bright field microscope image of the cross-section of the villus-like microstructured hydrogel scaffolds. Right: confocal maximum intensity projection showing F-actin and nuclei staining of Caco-2 cells grown on top of the villus-like scaffold. Scale bars: 100 μm.

The villus-like microstructured scaffolds were fabricated by photolithography, as previously described (Castaño et al., 2019) (Figure 1A). Briefly, a prepolymer solution containing 6.5% w/v 6 kDa PEGDA, 0.3% w/v acrylic acid (AA), and 1% w/v Irgacure D-2959 photoinitiator in phosphate-buffered saline (PBS) (all from Sigma-Aldrich) was flown into a chip fabricated with a 1 mm thick polydimethylsiloxane (PDMS) (Sylgard 184, Dow Corning) stencil containing an array of pools of 6.5 mm diameter. Silanized glass coverslips or Tracketch polyethylene terephthalate (PET) membranes of 5 μm pore size (Sabeu GmbH & Co.) were used as substrates. The microstructured PEGDA-AA scaffolds were fabricated by exposure to UV light under patterned photomasks with transparent windows of 100 μm in diameter and a density of 25 windows/mm^2^. The photolithography was performed in an MJBA mask aligner (SUSS MicroTech) using a power density of 25 mW/cm^2^. The prepolymer solution was exposed for 140–220 s to form the villus-like micropillars (Figure 1B). For the samples fabricated on glass coverslips, a second exposure of 15 s was performed to form a hydrogel base holding the microstructures together. After UV exposure, unreacted polymer and photoinitiator were washed out with PBS and the hydrogels were kept submerged at 4°C for at least 3 days to reach equilibrium swelling. Samples fabricated onto PET membranes were assembled on modified Transwell inserts using double-sided pressure-sensitive adhesive rings as detailed in Castaño et al. (2019). After swelling, and to provide the scaffolds with cell-adhesion motifs, the PEGDA-AA hydrogels were functionalized with 0.01% w/v collagen type I (Sigma Aldrich) via an *N*- (3-Dimethylaminopropyl)-*N*′-ethylcarbodiimide (EDC)/*N*-Hydroxysuccinimide (NHS) (Sigma Aldrich) mediated coupling.

Caco-2 cells (ATCC HTB-37) from passages 75–85 were expanded and maintained in 75 cm^2^ flasks containing DMEM-Glutamax medium (Gibco, Thermofisher), supplemented with 10% v/v fetal bovine serum (Gibco, Thermofisher), 1% v/v penicillin/streptomycin (Sigma-Aldrich), and 1% v/v non-essential amino acids (Gibco, Thermofisher). Cells were kept in an incubator at 37°C and 5% CO_2_ and passaged weekly. Cells were seeded in the villus-like scaffolds at a density of 2.5 × 10^5^ cells/cm^2^ and cultured for 21 days while changing media every other day. At different time points during culturing, cells were fixed with 10% neutral buffered formalin solution (Sigma-Aldrich) at RT for 30 min and kept in PBS at 4°C until further sectioning.

### Embedding Microstructured Hydrogels With Low Molecular Weight PEGDA

**FIGURE 2:**
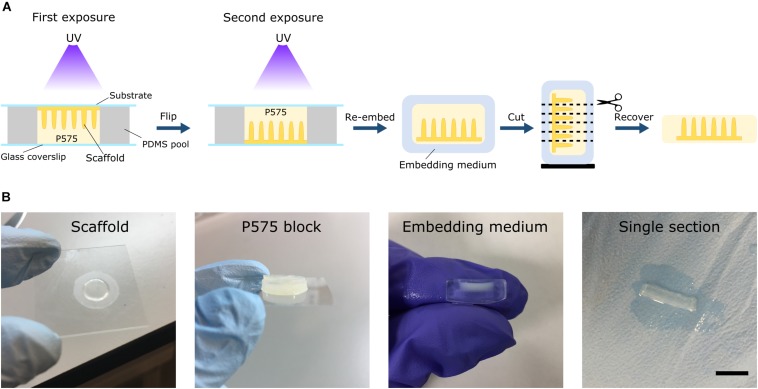
**(A)** Schematic drawing of the embedding protocol using low molecular weight PEGDA (P575) for soft hydrogel scaffolds with high aspect-ratio features. The scaffolds can be re-embedded in routine embedding media and sectioned using either cryosectioning or vibrating microtomy. **(B)** Image sequence of the different stages of the process. From left to right: a microstructured scaffold fabricated on PET membrane, the P575 block embedding the microstructured scaffold, the P575 block re-embedded in an agarose medium, and a free-standing, robust single section after vibrating microtomy sectioning. Scale bar: 5 mm.

The embedding medium was prepared by dissolving 10% w/v 575 Da PEGDA (P575) (Sigma-Aldrich) and 1% w/v Irgacure D-2959 photoinitiator in PBS. The solution was mixed at 65°C for 1 h, filtered and stored at 4°C until further use. Prior to light-induced embedding, microstructured scaffolds were incubated with the embedding medium overnight at 4°C. To facilitate the formation of P575 blocks, a PDMS chip was made. Briefly, the PDMS prepolymer was mixed with the curing agent in a 10:1 w/w ratio and degassed under vacuum. Then, it was cast to 4-well rectangular culture dishes (Thermo Fisher Scientific) to obtain PDMS slabs of 2 mm in height, which were cured for at least 2 h at 65°C. A pool of 10 mm in diameter was carved into the PDMS using a 10 mm diameter punch (Acuderm). Then, the pool was placed on top of a 24 mm × 60 mm glass coverslip (Menzel-Glaser). The pool was filled with the P575 embedding solution and the microstructured hydrogel samples grown on the glass coverslips or on the PET membranes (cultured with cells or not) were placed onto the pool upside down. The P575 embedding medium was then crosslinked by UV exposure using a UV lamp source (Mask aligner SUSS MicroTec MJB4 calibrated to a constant intensity of 25 mW/cm^2^ at 365 nm). First, the chip was irradiated for 100 s on one side and then flipped and exposed again for 100 s on the other (Figure 2A). For the hydrogel scaffolds prepared on PET membranes, a hydrogel support base was formed by first exposing 10% w/v P575 solution to UV for 40 s. The samples on the membrane were placed onto this support base and embedded in P575, as explained above. With this procedure, translucent blocks of P575 with the microstructured PEGDA-AA scaffolds inside were obtained (Figure 2B). The samples were kept in PBS at 4°C until sectioning, remaining unaltered for at least 1 month.

### Hydrogel Sectioning

For vibrating microtome sectioning, the P575 blocks were re-embedded in agarose to provide structural support. This second embedding medium was prepared by dissolving 8% w/v of low melting point agarose (CONDA) in PBS, boiling it twice in a microwave oven, and shaking it at 65°C for at least 30 min. A cryomold (Tissue-Tek) was filled halfway with the agarose solution and the P575 block was placed inside, orienting the sample properly for the desired section. Then, additional agarose solution was used to completely fill the mold. The agarose block was solidified at 4°C for 4 min, affixed to the specimen holder with Loctite super glue adhesive, and placed within a Leica VT1000S vibrating blade microtome, while ensuring that the tips of the villus-microstructures were facing the blade. The buffer tray bottom was filled with ice-cold PBS and the temperature was maintained by applying ice cubes to the outer holding chamber. The microtome was operated at the following parameters: 0.075 mm/s sectioning speed, 81 Hz oscillation frequency, 1 mm amplitude, and 300–350 μm section thickness. The cuts were recovered from the bath and kept in PBS at 4°C until further use, remaining unaltered for at least 1 month.

For cryotome sectioning, the P575 blocks were cut in half and incubated with OCT (VWR) overnight at 4°C. Thereafter, samples were transferred to cryomolds filled with OCT, frozen with liquid nitrogen and ice-cold 2-methylbutane (Sigma-Aldrich), and stored at −80°C. Histological cuts of 7–10 μm were obtained using a Leica CM1950 cryostat and air-dried for 15 min. Samples were stored at −20°C until further use, remaining unaltered for at least 1 month.

### Microstructural Assessment of Embedded and Sectioned Microstructured Hydrogels

To evaluate the morphology of the hydrogel villus-like microstructures after the embedding and sectioning processes, microstructured PEGDA-AA hydrogels were selectively stained by taking advantage of the flanking carboxylic groups from the acrylic acid polymer. For that, two strategies were used: Alcian Blue staining and functionalization with a fluorescent protein. Alcian Blue is a dye that has an affinity for acidic elements, such as the carboxylic acid residues of the PEGDA-AA hydrogels, so it should selectively stain the microstructured scaffold but not the embedding medium, which does not contain acrylic acid. The sections were incubated in a solution of 1% w/v Alcian Blue 8GX (Sigma-Aldrich) in 3% v/v acetic acid for 10 min at RT. Then, the sections were washed thoroughly with tap water for at least 10 min. Sections were visualized using bright field microscopy (Nikon Eclipse Ts2). Additionally, the flanking carboxylic groups of the microstructured hydrogels were functionalized with bovine serum albumin conjugated with Texas Red dye (BSA-TxRED) (Invitrogen) by EDC/NHS crosslinking chemistry. The vibratome sections were submerged into the EDC/NHS solution and incubated for 1 h at RT. Subsequently, the hydrogels were washed with PBS and incubated with 500 μg/mL BSA-TxRED solution in PBS for 1.5 h at RT. Then, the sections were washed again with PBS and visualized using a confocal laser scanning microscope (LSM 800, Zeiss).

### Immunofluorescence Staining

In order to prove that the standard cell immunostaining protocols can be applied to the tissue sections obtained with the P575 embedding medium, we used fixed cell samples of Caco-2 cells grown on microstructured PEGDA-AA hydrogels as described in Section “Fabrication of Villus-Like Microstructured Hydrogels and Cell Culture.” Antigen retrieval was performed in the recovered sections by boiling them in 10 mM citrate buffer and 0.05% v/v Tween 20 at pH 6.0 for 10 min in a microwave oven. Then, the cells were permeabilized with 0.5% v/v Triton X-100 (Sigma-Aldrich) for 30 min and blocked with a buffer containing 1% w/v BSA (Sigma-Aldrich), 3% v/v donkey serum (Millipore), and 0.2% v/v Triton X-100 in PBS for at least 3 h. The sections were then incubated with the primary and secondary antibodies for 72 and 24 h, respectively. Increased antibody incubation times allowed better penetration of large antibody molecules through the hydrogels. The primary antibodies and their dilutions were: mouse anti-villin (5 μg/mL, Abcam ab201989), rabbit anti-β-catenin (2 μg/mL, Abcam ab2365), mouse anti-β-catenin (2.5 μg/mL, BD Biosciences 610154), and rabbit anti-*zonula occludens* 1 (ZO-1) (2.5 μg/mL, Invitrogen 40-2200). As secondary antibodies, Alexa Fluor^®^ 488 donkey anti-mouse (Invitrogen A-21202), Alexa Fluor^®^ 647 donkey anti-rabbit (Jackson ImmunoResearch 111-607-003), and Alexa Fluor^®^ 568 donkey anti-goat (Invitrogen A-11057) diluted at 4 μg/mL, were used. The primary and secondary antibodies were diluted in a working buffer containing 0.1% w/v BSA, 0.3% v/v donkey serum, and 0.2% v/v Triton X-100 in PBS. Nuclei were stained with 4′, 6-diamidino-2-phenylindole (DAPI) (5 μg/mL, Invitrogen D1306) for 1–2 h. For the staining of filamentous actin (F-actin), no antigen retrieval was performed as it interfered with the staining. Therefore, after the sections were recovered, samples were permeabilized and blocked as explained above. Then, samples were incubated with Acti-stain 535 Phalloidin (100 nM, Tebu-bio) for 2 h and counterstained with DAPI for 1 h. All the incubations were performed at 4°C under shaking.

### Image Acquisition

For imaging, histological sections obtained with the vibrating microtome were mounted with PBS, whereas cryosections were mounted using the standard mounting medium Fluoromount-G (Thermo Fisher Scientific). Fluorescence images were acquired at randomly selected locations using either a confocal laser scanning microscope (LSM 800, Zeiss) with 10x dry (N.A. = 0.3), 20x dry (N.A. = 0.8), and 40x glycerol (N.A. = 1.3) objectives or a super-resolution inverted confocal microscope (LSM 880 – Airyscan Elyra PS1, Zeiss) in fast airyscan mode and with 63x oil (N.A. = 1.4) objective. The laser excitation and emission light spectral collections were optimized for each fluorophore, especially for the four-color scans, where the emission bands were carefully adjusted to avoid overlapping channels. The pinhole diameter was set to 1 Airy Unit (AU). For all images acquired, the optimal z-step was used as indicated by the equipment software. The bright field images were obtained by an inverted optical microscope (Eclipse Ts2, Nikon).

### Image Analysis

Confocal microscopy z-stacks were processed using ImageJ software (NIH). All images were corrected for brightness and contrast while maximum intensity projections, or single focal planes, were represented as indicated in the figure legends. For the z-stacks from immunostainings, a background subtraction with a rolling ball radius of 150 pixels and a median filter with a radius of 3 pixels were applied to ZO-1 and β-catenin channels in order to remove the background noise.

To estimate the height of the cells, intensity profile plot measurements along line segments orthogonal to the cell walls were performed using the F-actin staining. For each sample, each villus-like micropillar to be analyzed was selected and rotated into an upright vertical position with the central axis of the pillar being parallel to the y-axis. The single focal plane that provided the best segmentation of the cellular membrane by visual inspection was selected from the acquired z-stacks. To mitigate the effect of noise in the images, the profiles were endowed with a width of 10 pixels to average the intensity across (ImageJ Plot Profile function). The cell height was estimated as the distance between the two most significant intensity maxima found along the intensity profile. The intensity maxima were required to have a minimum significance of 5–10 grayscale levels (i.e., the minimum altitude of the maxima with respect to the two adjacent valleys). This process was assisted by an ImageJ macro where the only manual step was adjusting the line segments. The (x, y) coordinates of the line segments (by default taken from the center) were also recorded. The cell heights were plotted as a function of the normalized position along the vertical axis (y-axis) of the villus (0: base, 1: tip). A total of 386 cells in 21 pillars were analyzed for samples obtained from two independent experiments with two technical replicas each (*N* = 2, *n* = 2).

Cell nuclei were segmented from single focal planes extracted from the z-stacks acquired from DAPI staining. Micropillars were individually oriented into a vertical position, as above, by rotating the image accordingly. Then, an ImageJ macro was used for implementing the following steps: (1) forcing very low values (background) to zero, (2) filtering the image by Laplacian of Gaussian filter with a radius adapted to the characteristic size of the nuclei, (3) detecting the nuclei as regional intensity minima (user-defined detection tolerance), (4) editing the detected nuclei markers manually, (5) performing watershed segmentation from these markers on an edge detected version of the original image (magnitude of intensity gradient after slight Gaussian filtering) and, (6) analyzing connected particles in the resulting binary mask while keeping the particles within a user-defined area range (25 to 300 pixels). The measurements of the connected particles (nuclei) included the position (centroid) as well as the major and minor axes of the ellipses fitted to the particles. The aspect ratio (elongation) of nuclei was determined from the ratio of the major to the minor axis of the fitted ellipses. The cell nuclei aspect ratios were plotted as a function of the normalized position along the villus axis. A total of 473 cell nuclei in 11 micropillars were analyzed.

The orientation of the cell nuclei was also estimated from the single focal planes. First, the micropillars were individually oriented into a vertical position and toward the top of the image by rotating the image accordingly. Next, the major axes (direction of orientation) of several nuclei were manually marked by drawing line segments. Then, another line segment was drawn along the orientation of the hydrogel surface in the vicinity of the nuclei. The angle (0–90°) between these two-line segments was calculated using an ImageJ macro. The measurements also report the nuclei locations as the rescaled villus axis (0: base, 1: tip). The orientations of the nuclei (expressed as the angle), with respect to the hydrogel surface, were plotted as a function of the normalized position along the villus. A total of 440 cell nuclei in 11 micropillars were analyzed.

### Statistical Analysis

Graphs were plotted using GraphPad software. The data are presented in the figures as the mean ± standard error of the mean (SEM). The error bars represent the SEM of at least two independent experiments with two technical replicas. Statistical comparisons were performed using ANOVA test and *p*-values < 0.05 were considered to be significant.

## Results

### Poly(ethylene) Glycol Diacrylate Embedding Preserves the Structural Integrity of Villus-Like Hydrogel Scaffolds During Sectioning

3D villus-like hydrogel scaffolds were fabricated by lithography-based dynamic photopolymerization of PEGDA-AA prepolymer solutions, as described in Castaño et al. (2019). The microstructures were fabricated either on glass coverslips or on flexible porous PET membranes ready to be assembled into Transwell insert supports. The villus-like micropillars were 480 ± 40 μm in height and there were 2330 ± 30 micropillars/cm^2^. After reaching equilibrium swelling, the hydrogel scaffolds were functionalized with Collagen-I via EDC/NHS chemistry. Caco-2 cells seeded on top of the hydrogels covered the micropillars by forming a monolayer. After 3 weeks of culturing, cells were fixed and sectioned.

The villus-like microstructured hydrogels were embedded in 10% w/v low molecular weight PEGDA, P575, by UV-initiated crosslinking (Figure 2A). The formed P575 block embedded the finger-like structures in a conformal manner, providing the delicate structures with good mechanical stability. Then, the P575-embedded microstructured hydrogels were successfully re-embedded in routine embedding media (e.g., OCT and agarose) for cryosectioning or vibrating microtomy. In vibrating microtomy, minimum damage is made to fragile tissues as it does not require dehydration or freezing steps. However, the sections obtained by this technique are thick (≥50 μm) (Short et al., 2017). The P575-embedded hydrogel sections recovered from the vibratome were mechanically robust, which enabled further manipulation, immunostaining and imaging procedures (Figure 2B). On the contrary, sectioning the microstructured hydrogels with the vibratome was not possible without the P575 embedding. Furthermore, the standard embedding medium, agarose, did not sufficiently penetrate the hydrogel network to hold it within the block (Supplementary Figure 1). On the other hand, sectioning scaffolds fabricated onto porous membranes with vibrating microtomy was not possible, even after the P575 embedding. The difference in stiffness between the soft hydrogel and the hard PET membrane resulted in the sample being dragged by the blade, which ruined it. Instead, the scaffolds on membranes were processed with the cryotome. In cryosectioning, very thin tissue sections (5–15 μm) can be obtained, allowing for the visualization of fine details of the cells (Fischer et al., 2008). The P575 blocks containing the villus-like microstructures were embedded in OCT after overnight infiltration (Ruan et al., 2013). Cryosection sections of these samples were then successfully recovered on glass slides and were suitable for further processing.

**FIGURE 3:**
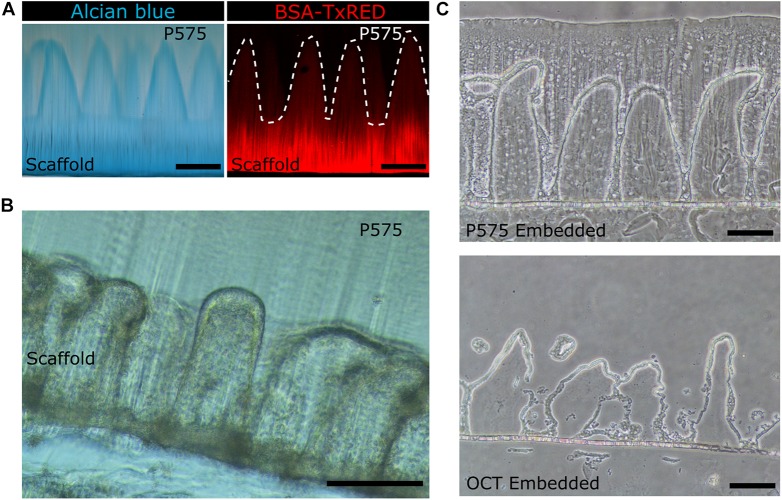
**(A)** Bright field microscopy image of villus-like microstructured PEGDA-AA hydrogels stained with Alcian Blue (left panel) and confocal maximum intensity projection image of the PEGDA-AA microstructures functionalized with BSA-Texas Red (right panel) after P575 embedding and sectioning. White dashed lines mark the border of the microstructures. Scale bars: 200 μm. **(B)** Bright field microscopy image of a vibratome section of the P575 embedded villus-like hydrogel scaffold with Caco-2 cells grown on top. Scale bar: 200 μm. **(C)** Bright field microscopy images of cryosectioned villus-like hydrogel scaffolds with Caco-2 cells grown on top. The upper panel shows the hydrogel embedded in P575 and then in OCT; the lower panel shows the control only with the OCT embedding. Scale bars: 100 μm.

To evaluate if the P575 embedding, and further sectioning steps, affected the morphology of the villus-like microstructures, vibratome sections were stained with Alcian Blue and fluorescently labeled BSA proteins. Both molecules bind selectively to the flanking carboxylic groups from the acrylic acid polymer and therefore allows the morphology of the micropillars (made of PEGDA-AA) within the P575 embedding block to be properly distinguished. Figure 3A shows that the villus-like features of the hydrogels were unaffected by the P575 embedding and further sectioning. In addition, the BSA fluorescence images demonstrate the suitability of the embedding medium for the staining with large proteins (Figure 3A, right panel).

Then, the compatibility of the embedding method with sectioning samples covered by epithelial cells was also tested. Figure 3B shows that vibrating microtomy sections of villus-like microstructured hydrogels, with an epithelial cell monolayer grown on top, can be obtained without damaging the structural integrity of the microstructures. Such structural integrity was also observed for the samples fabricated on porous membranes and used in cryosectioning. In this case, compared to the control sections, the P575 embedding significantly improved the preservation of the villi-like micropillars (OCT embedding) (Figure 3C and Supplementary Figure 2). More importantly, it should be noted that the sections obtained using the novel embedding technique were fully compatible with standard immunostaining and imaging procedures.

### High Magnification Imaging Reveals the Impact of Villus-Like Topography and Local Curvature on Intestinal Epithelial Cell Polarization and Orientation

**FIGURE 4:**
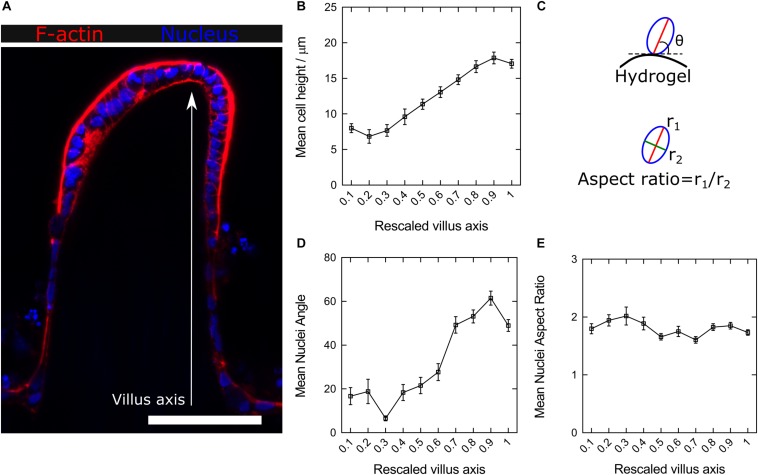
**(A)** Confocal microscopy image of F-actin and nuclei staining of the Caco-2 monolayer formed on top of microstructured hydrogels after 21 days in culture. Scale bar: 100 μm. **(B)** Average cell heights along the rescaled villus axis. **(C)** Schematic drawing showing the nuclei orientation (the major axis angle of the fitted ellipse with respect to the hydrogel surface) and elongation (the ratio between the major to the minor axes of the fitted ellipse) analysis. **(D)** Change in the mean nuclei angle (nuclei orientation) and **(E)** nuclei aspect ratio (nuclei elongation) along the rescaled villus axis. Data presented as mean ± standard error of the mean (SEM).

Cell–cell and cell-substrate interactions determining cell fate are affected by substrates containing 3D curved features with micron-scale dimensions (Assoian et al., 2019; Baptista et al., 2019). Previously, we have demonstrated that the villus-like 3D topography of PEGDA-AA hydrogels affects cell growth and improves the functional performance of the epithelial monolayers as tissue barriers, representing better tissue physiological permeability (Castaño et al., 2019). To explore the origin of such an improvement, histological sections of the scaffolds were obtained and imaged at high magnification by employing the new embedding method described here (Figure 4A and Supplementary Video 1). The Caco-2 monolayers grown on the 3D microstructures were stained for filamentous actin (F-actin) and DAPI at different time points during culturing. After 1 week of culture, cells covering the microstructures exhibited a cuboidal morphology but without a pronounced apicobasal polarity. After 2 weeks, and especially after 3 weeks of culturing, cells showed the columnar morphology characteristic of the differentiated intestinal epithelium (Supplementary Figure 3). After 3 weeks of culture, cell and nuclei morphology were quantitatively evaluated along the villi vertical axis and correlated with their position with respect to the base of their corresponding villus. High magnification images (Supplementary Figure 4) provided the necessary resolution for both proper cell segmentation and quantitative analysis of these cell features. Through these measurements, changes in the cell height, nuclear aspect ratio (elongation), and orientation along the vertical axis of the villi were revealed. Cell height, determined from the F-actin staining, significantly increases by more than twofold, from 7 ± 4 to 18 ± 5 μm (mean ± SD), when measured from cells located at the bases of the villi to the cells located at their tips (Figure 4B). On the other hand, cell nuclei orientation, expressed as the angle θ between the major axis of an ellipse fitting nucleus shapes and the tangent to the hydrogel surface at the place where each nucleus is located (Figure 4C, upper panel), were also quantified. It was found that the nuclei of cells located on the lateral surfaces of the villus-like structures, which have a planar topography, were mostly oriented parallel to these surfaces (θ < 20°, Figure 4D). Conversely, the nuclei of cells located closer to the regions of maximum surface curvature (close to the tips) exhibited orientations which are significantly more perpendicular to the surface (θ > 60°) (Figure 4D). An additional parameter often related to epithelial polarization, i.e., the elongation of cell nuclei, was also calculated along the villus-like axis (base to tip) of the different microstructures analyzed. The obtained values, expressed as the nuclei aspect ratio between the major and the minor axes of the fitted ellipse (Figure 4C, lower panel), did not change significantly along the vertical axis of the villi. Remarkably, all the cell nuclei analyzed were elliptically elongated with values ranging from 1.6 to 2.0, where 1 being the value of a circle (Figure 4E).

**FIGURE 5:**
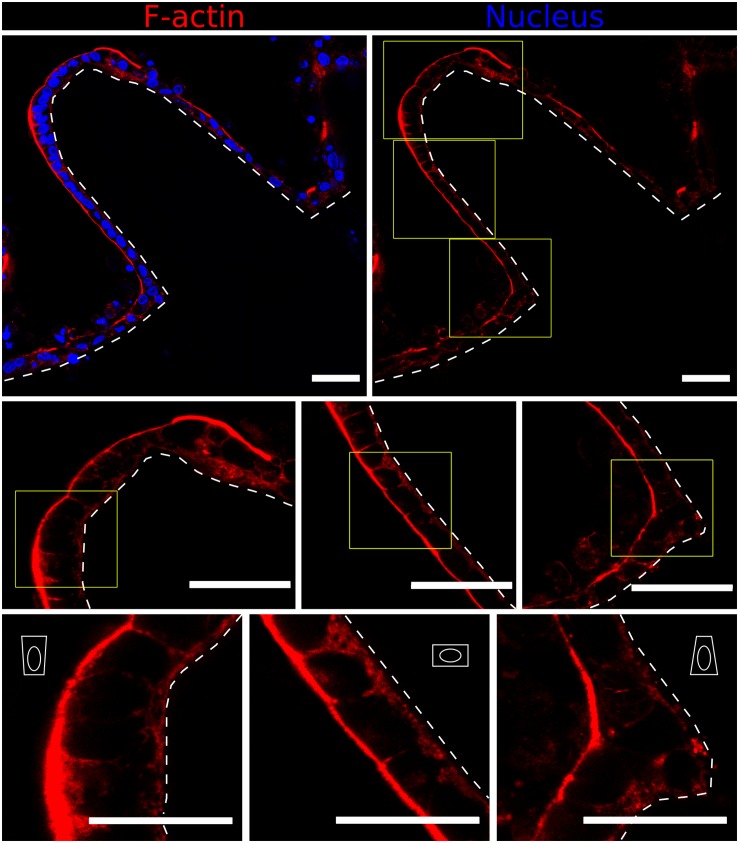
Fast Airyscan confocal images showing F-actin and nuclei staining of the Caco-2 monolayer formed on top of microstructured hydrogels after 21 days in culture (upper row). Yellow boxed regions are shown in high magnification (middle and lower rows). Different cell shapes are observed: basally constricted wedge-shaped at the villi tips, cuboidal along the walls and apically constricted at the base, as represented by the white drawings. Scale bars (upper and middle rows): 50 μm, (lower row): 30 μm.

To further assess in greater detail the specific cell-morphology effects of the local curvature, high-resolution images were acquired using the fast Airyscan modality of a super-resolution inverted confocal microscope equipped with a 63x oil objective lens. Tile scans were performed to image the entire microstructure (Figure 5, upper row). High magnification images performed at precise locations displaying convex (tip apex), planar (lateral walls), and concave (base) curvatures showed a progressive change in cell morphology which adapts to the curvature of the hydrogel surfaces (Figure 5, middle row). Cells were found to be basally constricted and thus wedge-shaped at the convex surfaces (villi tips), possessed a cuboidal morphology on the planar surfaces (villi walls), and be apically constricted at the concave surface of villi bases (Figure 5, lower row), revealing differential apicobasal tensions along the villus-like microstructures. Altogether, these results demonstrate that cells on the villus-like structures were all polarized (nuclei elongated, accumulation of actin at the apical side), but that their polarization state (according to cell height, nuclei orientation, visual differences of apical actin and cell morphology) changes along the vertical axis of the villus-like microstructures, suggesting a good correlation with the local changes in surface curvature.

### High-Resolution Imaging of Intestinal Epithelial Cell Markers Suggests a Differential Expression Along the Vertical Axis of Villus-Like Structures

On the one hand, we had previously reported that Caco-2 cell monolayers grown onto 3D villus-like scaffolds of PEGDA-AA showed barrier properties that were more similar to physiological tissues than those grown onto standard 2D flat cell cultures (Castaño et al., 2019). On the other hand, the results presented in the previous section showed significant changes in epithelial cell height and morphology along the vertical axis of the villus-like structures. Therefore, this raised the question if the morphological changes observed correlated with the differential expression of epithelial markers, indicating that cell polarization and cell–cell tight junctions might be the origin of the improved barrier performance reported.

**FIGURE 6:**
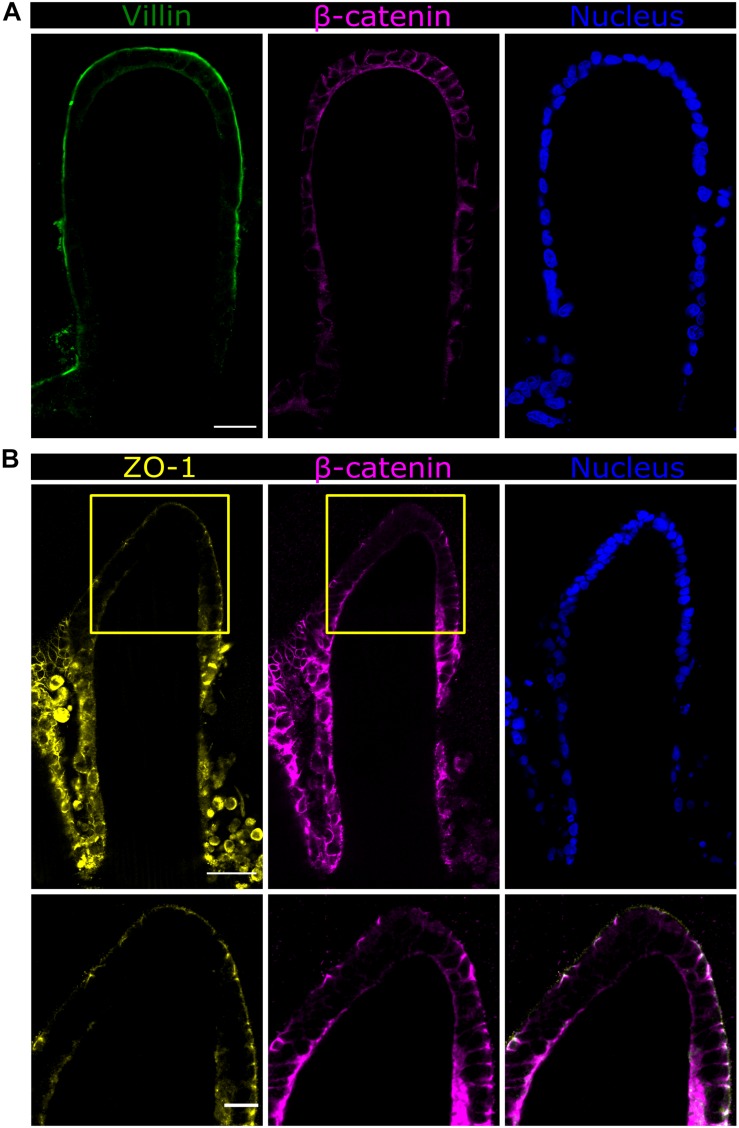
Fast Airyscan confocal images of the Caco-2 monolayer formed on top of the microstructured hydrogels after 21 days in culture showing the expression of **(A)** villin in green or **(B)** ZO-1 in yellow, β-catenin in magenta, and nuclei in blue. Yellow boxed regions are shown in high magnification. Scale bars (upper and middle rows): 50 μm, (lower row): 20 μm.

In order to visualize the intestinal epithelial cell marker distribution along the villus axis of the microstructures, vibratome sections of P575-embedded villus-like hydrogels were stained using a minimally adapted routine immunostaining procedure. Sections were then imaged at high resolution (63x magnification) and tile scans spanning the entire microstructure were acquired. Caco-2 cells, grown for 21 days on top of the villus-like scaffolds, expressed the epithelial cell markers located at their proper locations. Specifically, villin protein was found in the microvilli at the cell apical membrane while β-catenin was shown to accumulate in the lateral and basolateral cell membranes (Figure 6A). Remarkably, the expression of the tight junction protein z*onula occludens*-1 (ZO-1) was spatially encoded along the villus-like structures. Cells located at the tip of the villi showed ZO-1 localization at the apex of the lateral membrane (Figure 6B). This spatially coincides with the convex surfaces displaying the most columnar morphology, as determined in the previous section. More importantly, the images show ZO-1 exquisitely located at the top of the β-catenin staining (Figure 6B), just as in native intestinal epithelium (Zihni et al., 2016), thus also indicating the good polarization of the intestinal epithelial cells on the PEGDA-AA villi-like hydrogel scaffolds.

## Discussion

Over the last two decades, 3D culture models have been developed to provide cells with a more physiologically relevant *in vitro* environment with the aim to reduce the gap between two-dimensional cell cultures and live tissues for studying healthy and disease states as well as for preclinical drug screening (Pampaloni et al., 2007; Booij et al., 2019). The effect of surface topography on cell behavior has been explored in numerous studies where it was mostly conducted on single cells and focused on the cellular and subcellular scale (Martínez et al., 2009; Théry, 2010). However, most physiological occurring structures, such as glands, alveoli, or intestinal villi, have topographies in the range of hundreds of micrometers and they are inherently curved. Furthermore, there is increasing evidence that substrate topography and curvature on a micron-scale also affects cell fate and cell–cell interactions (Broaders et al., 2015; Yevick et al., 2015; Assoian et al., 2019; Baptista et al., 2019). In our previous study, we fabricated microstructured 3D hydrogel scaffolds containing finger-like protrusions mimicking the architecture of small intestinal epithelium (Castaño et al., 2019). We observed that, compared to flat monolayer cultures, the growth of Caco-2 intestinal epithelial cells on these villus-like microstructures of physiological dimensions improved both cell polarization and cell nuclei elongation (Castaño et al., 2019). However, due to the limitations of the optical microscopy methods, these analyses were performed over a collection of low magnification z-stack confocal images acquired along the villi-like structure.

At present, there is a need to adapt the conventional handling and imaging techniques developed for flat cultures to more suitable for 3D cultured microtissues (Montanez-Sauri et al., 2015; Booij et al., 2019). Microengineered 3D samples, such as these villus-like microstructures, are typically several hundreds of micrometers thick and yield high light scattering, thus limiting the imaging of the whole construct with standard optical techniques such as confocal fluorescence microscopy. Some of the limitations include a reduced penetration depth when using high numerical aperture lenses and photobleaching, which is induced by the illumination of the entire object (Smith et al., 2010). The histological sectioning of the engineered constructs might overcome some of these drawbacks. By obtaining thin cross-sections of the samples, high magnification imaging can be achieved while cell behavior and morphology can be evaluated along the sample vertical axis in a single focal plane. However, standard histology procedures should be adapted for the special characteristics of tissue-engineered scaffolds.

In the case of villus-like hydrogel scaffolds, these high aspect-ratio structures should be well-preserved and their water-rich nature and soft mechanical properties should also be considered. Since the paraffin wax embedding method is incompatible with these structures, we investigated the microstructure integrity after sectioning with a vibrating microtome or cryotome. Both methods require the specimen to be embedded into agarose or OCT, respectively. Several authors have optimized the embedding and sectioning protocols for tissue-engineered hydrogel samples (James et al., 2004; Yang et al., 2007; Ruan et al., 2013; Short et al., 2017). However, those materials were prepared in bulk, leading to 3D flat constructs with no complex topographical features. In the presence of such delicate structures, these methods were unsuitable as the villus-like micropillars were not well-preserved after sectioning. In one instance, the agarose embedding did not sufficiently penetrate to hold the microstructured sample in place and sectioning could not be performed. Instead, the agarose acted as a molding material, a strategy previously used in the literature for the fabrication of villus-like structures (Costello et al., 2014a, b). In the case of the samples processed by cryosectioning, after overnight incubation with OCT following the protocol by Ruan et al. (2013), thin sections could be obtained but the hydrogel pillars showed obvious shrinkage and the microstructures collapsed. Thus, we developed a method in which the 3D microstructured hydrogel was transformed into a non-structured hydrogel block that can further be embedded into the standard media for histological sectioning without the risk of collapsing the structures. The microstructures were embedded using a light-based approach and a low molecular weight P575 prepolymer, creating a hard hydrogel block after UV exposure. The short polymer chains penetrated the pores of the microstructured hydrogels and, upon polymerization, the topography and dimensions of the microstructures remained unaltered. We demonstrated that this embedding method can be applied to cell-free and cell covered scaffolds fabricated in different substrates, such as glass coverslips or porous membranes, and that it is compatible with vibratome or cryotome sectioning upon second embedding in the corresponding standard media. This versatility allows for the selection of an adequate method for the desired end-use application.

By using this novel embedding protocol, the longitudinal core section of the villus-like micropillars could be obtained and imaged via high-resolution, high-magnification confocal microscopy. Thus, we analyzed, in a spatially resolved manner, the cellular and nuclear morphology along the villi. Moreover, a clear difference was observed in cell height and shape at different regions of the villi that presented distinguished curvatures. Cells at the base experienced a negative curvature and exhibited a trapezoidal shape with an apical constriction. Cells then transitioned to the flat region of the villus, adopting a cuboidal shape. In addition, cell height steadily increased toward the tip where it reached a maximum in the section of the tip with maximum convex curvature. The evolution of cell height along the villus structures was in agreement with the mechanical model of epithelial folds (Štorgel et al., 2016) and the quantification of cell morphology along the crypt-villus axis of *ex vivo* intestinal tissue in mice (Krndija et al., 2019). The orientation of nuclei along the villi showed a significant change in the angle when cells were at the curved region of the villi (*x* > 0.6). Yu et al. (2018), also reported effects on cell height and alignment of renal epithelial cells when cultured on curved surfaces. In that study, they also observed an increased ZO-1 expression when cells were exposed to a highly concave curvature. For the villus-like microstructures reported here, polarization markers of intestinal epithelial cells, such as ZO-1 and β-catenin, were differentially expressed at the tip of the villi, again highlighting that the 3D curvature is crucial for cell morphology and differentiation. This differential expression along the vertical axis of the villus-like features might impact the permeability of the epithelial barrier formed and, thus, explains the experimental differences measured in the functional assays between cells grown on 3D curved surfaces and those grown on flat substrates (Castaño et al., 2019).

In summary, we examined the cell morphological response to villus complex topography in a microengineered hydrogel scaffold using a novel embedding method to preserve these complex features. This versatile photopolymerization-based method, compatible with both cryosectioning and vibrating microtomy, yields well-preserved microstructures that can be further analyzed with high-resolution and high-magnification microscopy. Analysis of the cellular and nuclear morphology of epithelial cells demonstrated that the topography and curvature along villus-like microstructures can have a significant effect. The combination of new sample processing methods, such as the one here described, with state-of-the-art 3D *in vitro* models would help to study the physiological events observed in *in vivo* models in a systematic and reproducible manner. This method would also be useful for *in vitro* models of morphogenesis, for mechanistic studies such as curvotaxis, as well as for pathological situations in which there are changes in the physiological structures. For example, some enteropathies such as celiac disease, present villus atrophy as the histopathologic hallmark, which has been related to defective intestinal permeability and an altered distribution of the tight junction proteins (Schumann et al., 2017; Jansson-Knodell et al., 2018). This work would significantly contribute to the widespread implementation of 3D microengineered tissues for those applications, ultimately leading to more effective discovery of new drugs.

## Data Availability Statement

The raw data supporting the conclusions of this article will be made available by the authors upon request.

## Author Contributions

GA performed the experiments and analyzed the data. ST developed the macros for image analysis. GA together with MG-D designed the experiments. MG-D provided hydrogel scaffolds cultured with Caco-2 cells. EM and MG-D coordinated the project and together with GA wrote the manuscript.

## Conflict of Interest

The authors declare that the research was conducted in the absence of any commercial or financial relationships that could be construed as a potential conflict of interest.
