# Supplementary material for: Imaging the Cell Morphological Response to 3D Topography and Curvature in Engineered Intestinal Tissues
Source: Front Bioeng Biotechnol. 2020 Apr 7;8:294. doi: 10.3389/fbioe.2020.00294 (PMC7154059; doi:10.3389/fbioe.2020.00294)
Supplement: Supplementary file 1 [file Table_1.DOCX]

Supplementary Material

**Imaging the cell-morphological response to 3D topography and curvature in engineered intestinal tissues**

Gizem Altay^1^, Sébastien Tosi^2^, María García-Díaz^1*^, Elena Martínez^1,3,4*^

^1^Biomimetic systems for cell engineering, Institute for Bioengineering of Catalonia (IBEC), The Barcelona Institute of Science and Technology (BIST), Barcelona, Spain

^2^Advanced digital microscopy core facility (ADMCF), Institute for Research in Biomedicine (IRB Barcelona), The Barcelona Institute of Science and Technology (BIST), Barcelona, Spain

**^3^**Centro de Investigación Biomédica en Red en bioingeniería, Biomateriales y Nanomedicina (CIBER-BBN), Madrid, Spain

^4^Department of Electronics and Biomedical Engineering, University of Barcelona (UB), Barcelona, Spain

*** Correspondence:**
María García-Díaz
mgarcia@ibecbarcelona.eu

Elena Martínez
emartinez@ibecbarcelona.eu

# Supplementary Figures


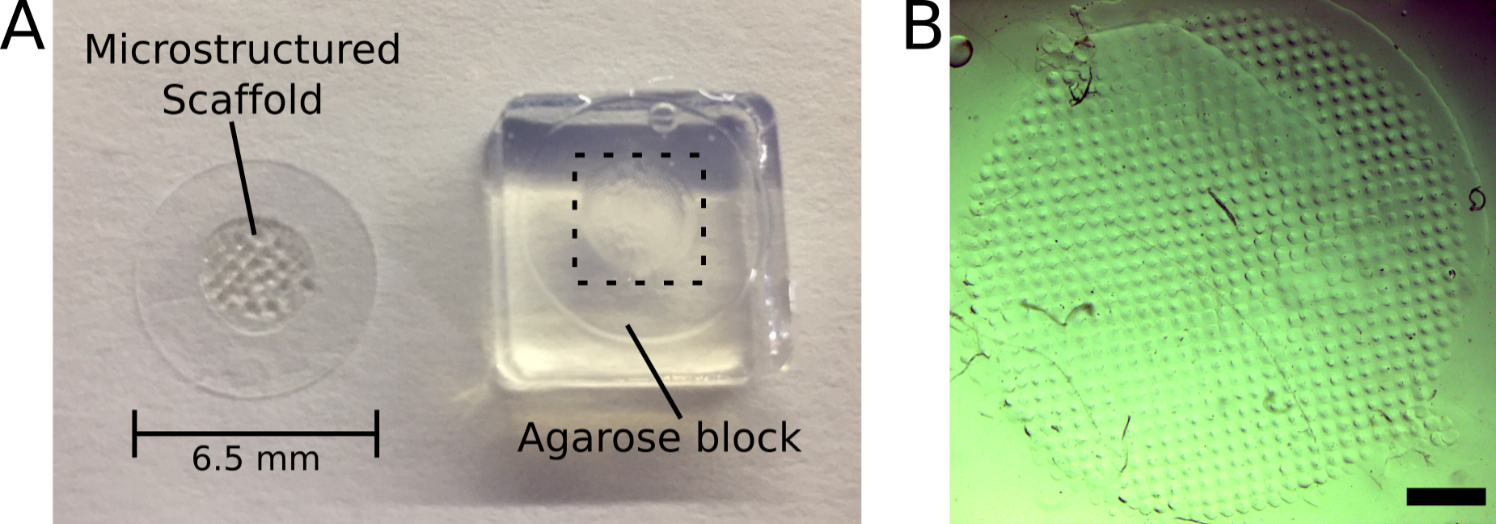


**Supplementary Figure 1.** **(A)** Image showing the microstructured scaffold fabricated on a glass coverslip (left) and the agarose block (right) after embedding the scaffold without the P575 pre-embedding. The agarose acted as a molding agent rather than an embedding media. **(B)** Bright field microscope image of the black boxed region showing microstructure forms patterned on the agarose block. Scale bar: 1 mm**.**

**
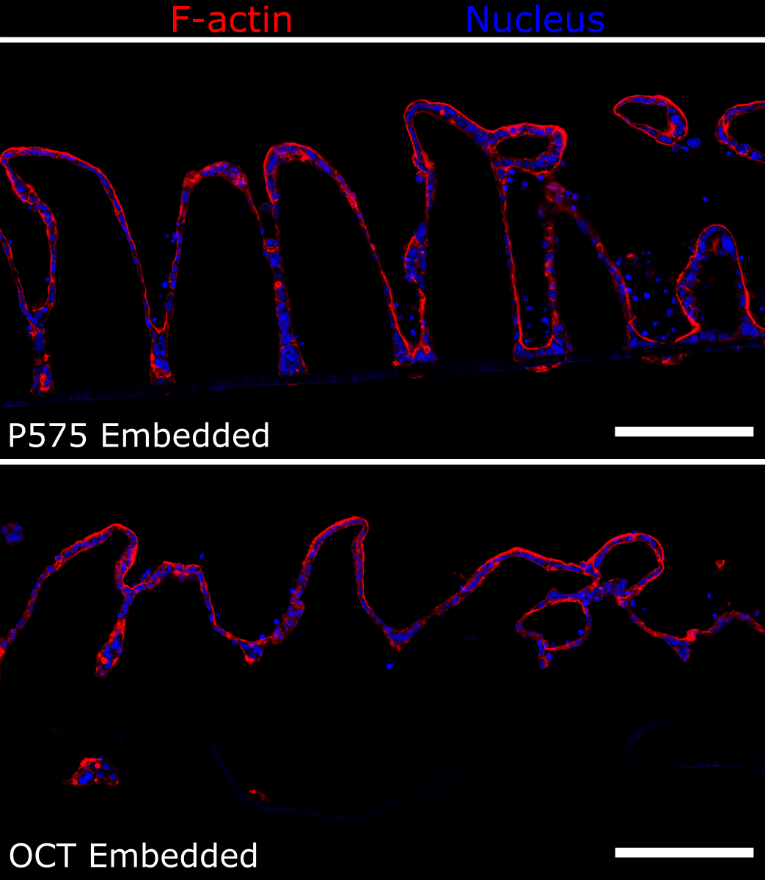
**

**Supplementary Figure 2.** Effect of P575 embedding on cryosections. Confocal images show F-actin and nuclei staining of the Caco-2 monolayer formed on top of microstructured hydrogels after 7 days in culture. Scale bars: 100 𝜇m.

**
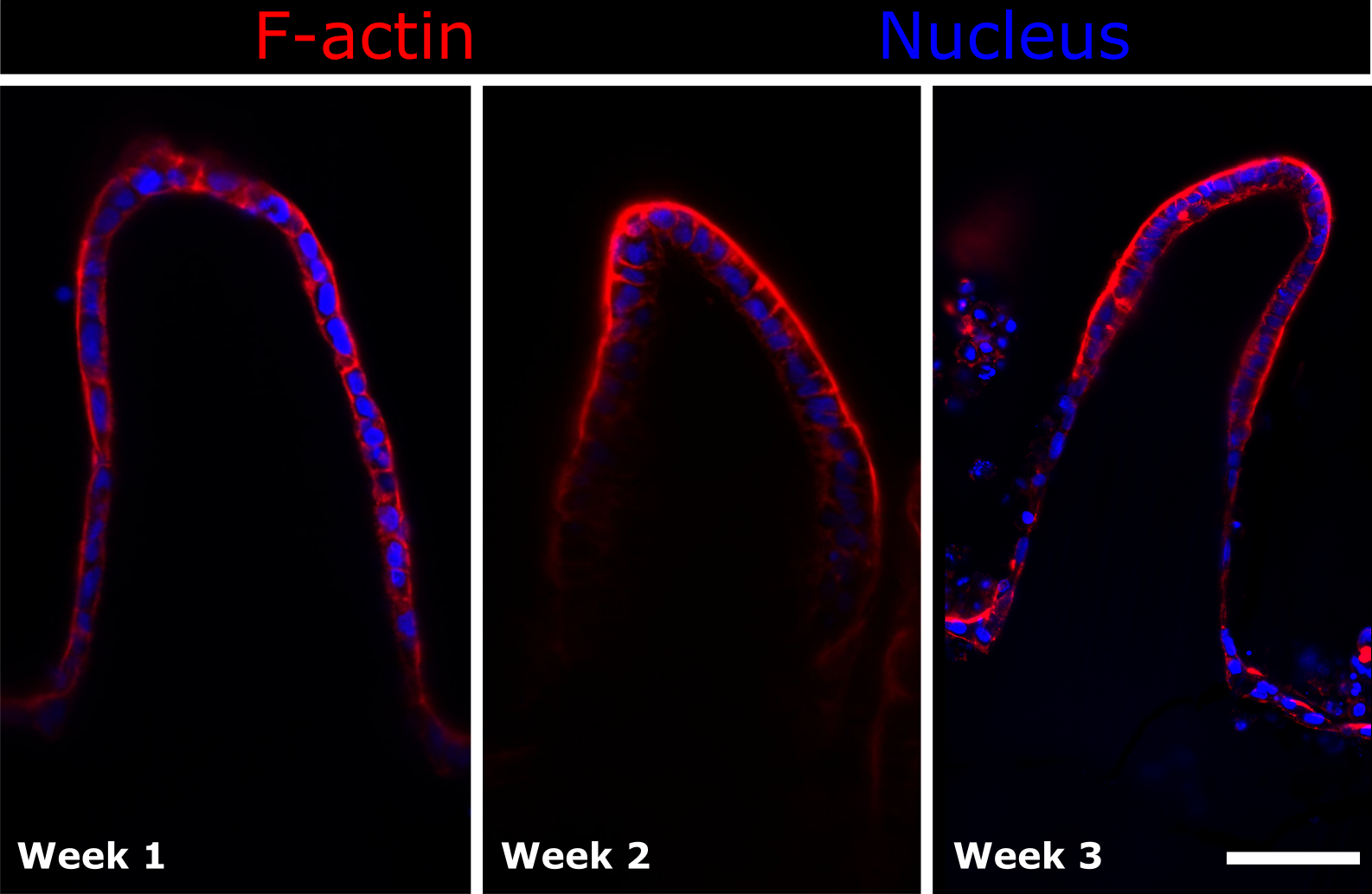
**

**Supplementary Figure 3.** Confocal microscopy images showing F-actin and nuclei staining of the Caco-2 monolayer formed on top of microstructured hydrogels at different timepoints during culturing. Scale bar: 100 𝜇m.


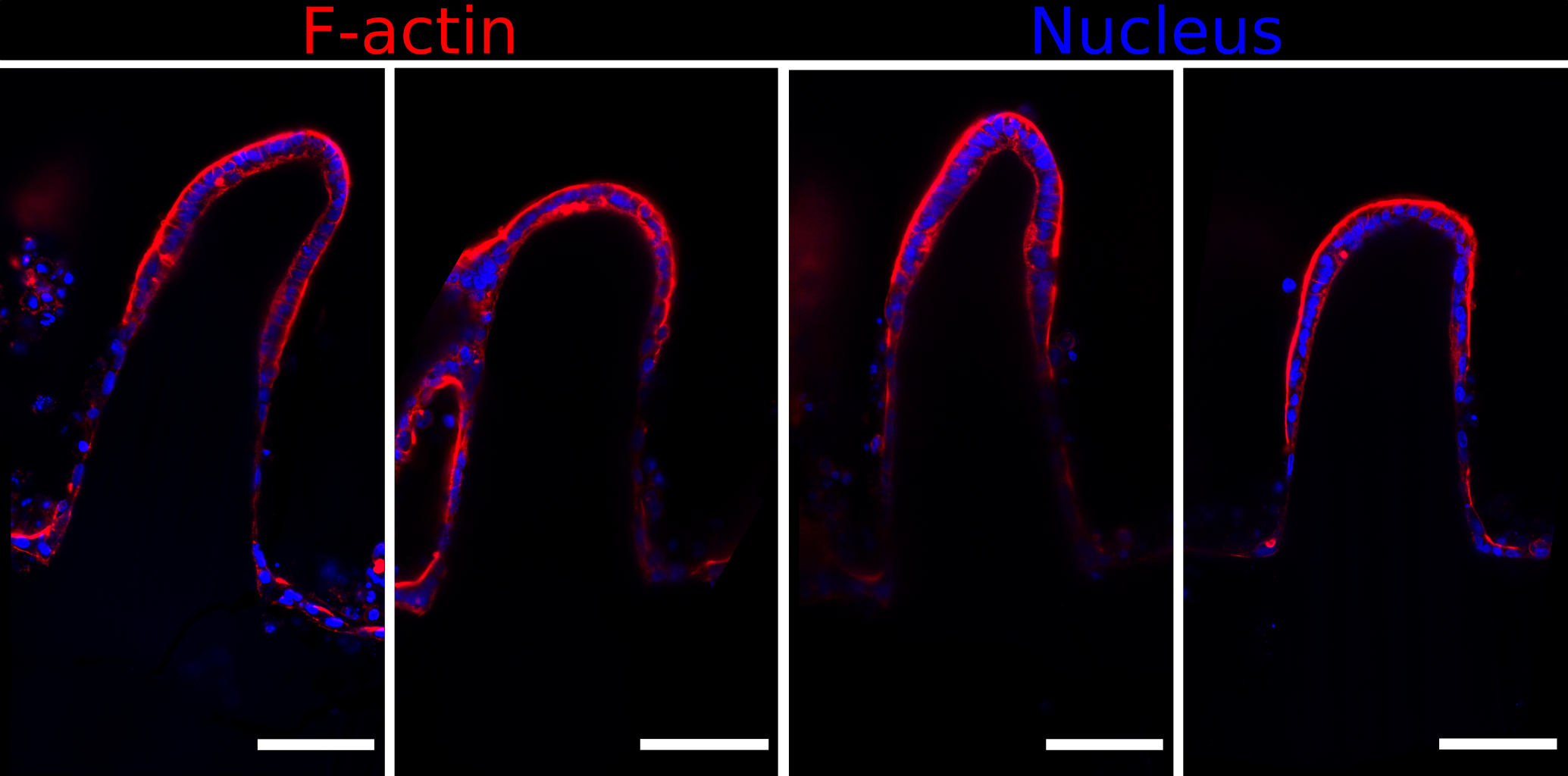


**Supplementary Figure 4.** Series of confocal microscopy images showing F-actin and nuclei staining of the Caco-2 monolayer formed on top of microstructured hydrogels after 21 days in culture. Different microstructures of the same condition are represented. Scale bars: 100 𝜇m.
